# A MEN1 Patient Presenting With Multiple Parathyroid Adenomas and Transient Hypercortisolism: A Case Report and Literature Review

**DOI:** 10.3389/fendo.2022.802453

**Published:** 2022-03-08

**Authors:** Fuqiong Chen, Qinqin Xu, Wenzhu Yue, Xuefeng Yu, Shiying Shao

**Affiliations:** ^1^ Division of Endocrinology, Tongji Hospital, Huazhong University of Science & Technology, Wuhan, China; ^2^ Branch of National Clinical Research Center for Metabolic Diseases, Wuhan, China

**Keywords:** multiple endocrine neoplasia type 1, hyperparathyroidism, hypercortisolism, parathyroid adenomas, hypercalcemia

## Abstract

**Background:**

Multiple endocrine neoplasia type 1 (MEN1) is a hereditary endocrine syndrome caused by mutations in MEN1 tumor suppressor gene.

**Case Presentation:**

A 53-year-old Chinese female was admitted to Division of Endocrinology, Tongji Hospital, for hypercalcemic crisis. Increased level of parathyroid hormone (PTH) was confirmed by laboratory tests, and imaging examination showed multiple parathyroid adenomas. Based on gene analysis, the patient was diagnosed as MEN1 associated hyperparathyroidism (HPT) by gene analysis with c.1378C>T (p.Arg460Ter) mutation in MEN1 gene. Her condition was complicated by transient hypercortisolism, mammary mass and uterine leiomyoma. After subtotal parathyroidectomy, PTH and serum calcium levels returned to normal.

**Conclusion:**

HPT with multiple parathyroid adenomas is an indication of MEN1 gene mutation. Serum cortisol and its circadian rhythm can be abnormal in the presence of hypercalcemia and high PTH. These parameters can return to normal after parathyroidectomy.

## Introduction

Multiple endocrine neoplasia (MEN) is characterized by the occurrence of two or more endocrine tumors in a single patient (1). There are four major types of MEN denoted as MEN1-4 (2). Each type of MEN is characterized by the occurrence of tumors in specific endocrine glands, inherited as an autosomal-dominant syndromes or may be sporadic (1).

The classic manifestation of MEN1 is co-occurrence of parathyroid, pancreatic islet, and anterior pituitary tumors. Other neoplasms may occur during the course of MEN1, including adrenal tumors, gastric tumors, skin and subcutaneous tumors, as well as breast cancer reported recently (3, 4). The incidence of MEN1 has been estimated to be 0.25% from randomly chosen postmortem studies, and to be 1-18% in patients with primary hyperparathyroidism (PHPT) (3). A diagnosis of MEN1 is established if a patient has one of three manifestations: 1) two or more main MEN1-associated endocrine tumors, 2) one MEN1-associated tumor and a first-degree relative of a confirmed MEN1 patient, 3) a germline mutation in MEN1 gene (3).

Here, we report a complex case of MEN1 associated with symptomatic PHPT and a transient hypercortisolism. Multiple parathyroid adenomas raised our concerns regarding the diagnosis of MEN1. To our knowledge, dynamic change of cortisol level in MEN1 patient has not been reported before.

## Case Description

A 53-year-old Chinese female was referred to local hospital in April 2019 because of her sore left knee (**Supplementary Figure 1**). After admission, the patient was diagnosed with bone cyst of the left patella and osteoporosis based on X-ray and dual-energy X-ray absorptiometry (DEXA) scanning. The preoperative examinations showed a significant increase in serum calcium (4.03mmol/L), while a decrease of potassium level (2.9mmol/L). Thus, an operation proposed based on the primary diagnosis was canceled. Although the patient received fluid infusion and potassium supplementation, the serum potassium and calcium concentrations did not return to normal levels. Subsequently, the patient was transferred to our department for further clinical evaluation. The patient suffered from dry mouth, fatigue, and muscular weakness in the past year. There was no nausea, poor appetite, back pain, neurological alterations, and other discomforts. She didn’t receive any medical treatment. In addition, the patient had a history of hypokalemia, hypertension and hysterectomy for uterine leiomyoma. She took anti-hypertension medications (calcium channel blockers) and oral potassium tablets intermittently. Her mother had a long history of hypertension and type 2 diabetes and her father had died of gastric cancer. There was no family history of electrolyte disturbances, psychosocial and hereditary disease.

On admission, the patients was conscious with a body temperature of 36.5°C, pulse rate of 96 beats/min, and blood pressure of 140/99mmHg. An oval-shaped mass with regular edges was palpable on the left side of the neck. There were no symptoms or signs of hypoglycemia, headache, vision loss, moon face, hirsutism, purple striae, or central obesity.

Laboratory examinations revealed notable elevations in parathyroid hormone (PTH) (1917.00 pg/mL, normal range 15-65 pg/mL) and calcium (4.14 mmol/L, normal range 2.15-2.50 mmol/L), indicating hypercalcemic crisis (**Table 1**). Based on these findings, the patient was diagnosed as PHPT. The patient also presented a hypercortisolism and loss of circadian rhythm (8am 221.00 µg/L, 4pm 287.70 µg/L, 12MN 281.40 µg/L, **Table 2**), while adrenocorticotrophic hormone (ACTH) level was normal. The serum cortisol could not be inhibited by low-dose overnight dexamethasone suppression test (DST) (**Table 2**) (5).

**Table 1:** Electrolyte and PTH levels before and after parathyroidectomy.

Day	-7	-5	-3	-1	0	1	3	10	76	137
PTH (15-65 pg/mL)	1917.00	2055.00	/	2942.00	/	76.96	29.88	/	219.60	153.50
Ca (2.15-2.5mmol/L)	4.14	3.83	2.54	2.54	2.60	2.26	2.35	2.32	2.22	2.31
K (3.5-5.1mmol/L)	3.06	3.61	3.20	3.13	3.52	3.60	3.10	5.32	4.03	3.60
P (0.81-1.45mmol/L)	1.00	0.85	0.45	0.54	/	/	0.68	0.79	0.89	/

Ca, Calcium; K, Potassium; P, phosphorus; PTH, Parathyroid hormone; Day 0, date of surgery; /, not detected.

**Table 2:** Laboratory examinations before and after parathyroidectomy.

		Pre-operation		Post-operation		1^st^ Follow-up	2^nd^ Follow-up
Blood routine tests	WBC (3.5-9.5X109/L)	9.77		5.45		/	/
	RBC (3.8-5.1X1012/L)	3.42		2.29		/	/
	Hb (115-150 g/L)	111		73		/	/
	PLT (125-350X109/L)	312		305		/	/
Liver function	ALT (≤33 U/L)	63		<5		/	/
	AST (≤32 U/L)	56		11		/	/
	ALP (35-105 U/L)	208		227		/	/
Renal function	BUN (2.6-7.5 mmol/L)	6.39		6		/	/
	Cr (45-84 µmmol/L)	135		136		/	/
	UA (142.8-339.2 µmol/L)	350		298		/	/
	eGFR ( >90 mL/min/1.73m2)	38.6		38.3		/	/
Glucose and insulin tests	FPG (4.11-6.05 mmol/L)	6.02		/		/	/
	FINS (1.8-11.8 µIU/mL)	17.6		/		/	/
Sex hormone	PRG (0.00-0.78 ng/mL)	2.06		/		/	/
	FSH (16.74-113.59 mIU/mL)	82.48		/		/	/
	LH (10.87-58.64 mIU/mL)	51.85		/		/	/
	PRL (2.74-19.64 ng/mL)	25.15		/		/	/
	Estradiol (≤40 pg/mL)	33		/		/	/
	Testosterone (≤0.75 ng/mL)	0.31		/		/	/
	β-HCG (≤8.3 mIU/mL)	0.46		/		/	/
ACTH and Cortisol		1mg DXM suppression		High dose DXM suppression			
		Before	After	Before	After		
	ACTH (1.6-13.9 pmol/L)	2.89	2.21	9.85	0.72	6.16	4.37
	8a.m. Cortisol (60.2-184 µg/L)	221	131	116.5	22.87	113	154.2
	4p.m. Cortisol (26.8-105 µg/L)	287.7	/	/	/	/	/
	12MN Cortisol (µg/L)	281.4	/	/	/	/	/
Renin and Aldosterone concentrations	Renin (4.4-46.1 µIU/mL)	/		119.4		64.4	/
	Aldosterone (0-353 pg/mL)	/		153		104	/
	ARR	/		1.3		1.6	/
Adrenal medullary hormone	Metanephrine (≤0.21nmol/L)	0.18		/		/	/
	Normetanephrine (≤0.59 nmol/L)	0.36		/		/	/
Others	GH (0-10 ng/mL)	0.92		/		/	/
	IGF-1 (255±85 ng/mL)	183		/		/	/
	25-hydroxy vitamin D (>30 ng/mL)	7.1		/		/	/

ACTH, adrenocorticotrophic hormone; ALP, alkaline phosphatase; ALT, alanine transaminase; ARR, aldosterone-renin ratio; AST, aspartate transaminase; BUN, urea nitrogen; Cr, creatinine; DXM, dexamethasone; FPG, fasting plasma glucose; FINS, fasting insulin; FSH, follicular stimulating hormone; GFR, glomerular filtration rate; GH, growth hormone; Hb, hemoglobin; HCG, human chorionic gonadotropin; IGF-1, Insulin-like growth factor 1; LH, luteinizing hormone; PLT, blood platelet; PRL, prolactin; PRG, progesterone; RBC, red blood cell; UA, uric acid; WBC, white blood cell; /, not detected.

**Figure 1:**
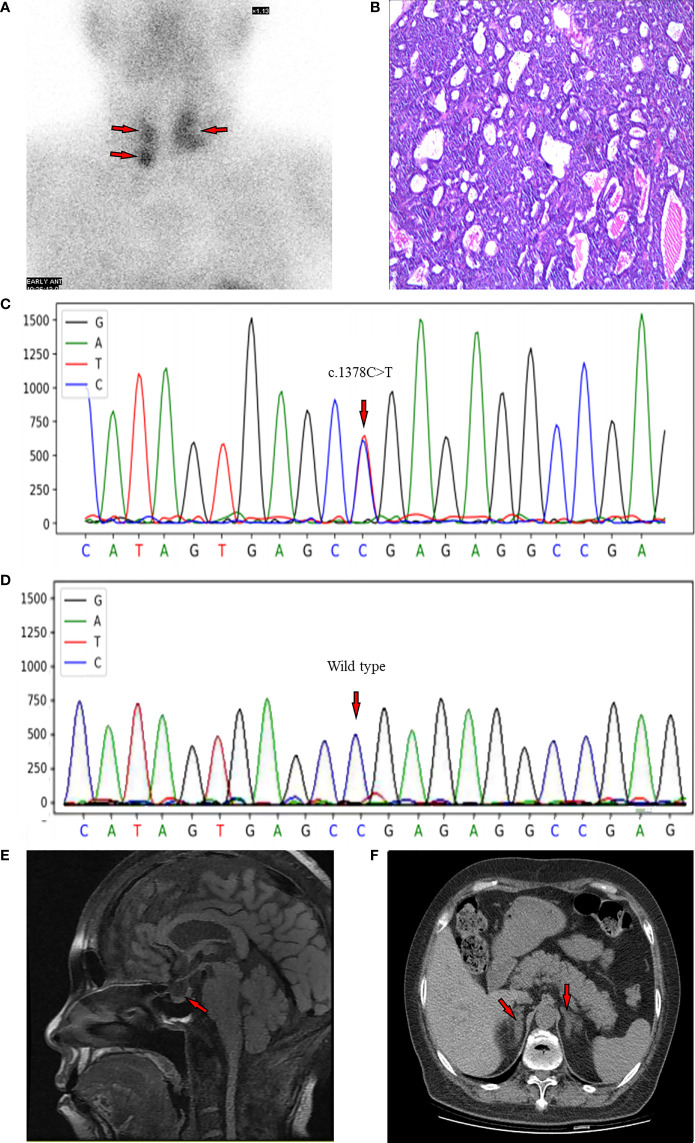
**(A)** MIBI scan of the parathyroid with three focal uptakes. **(B)** Histopathologic image of tissue parathyroidectomy. **(C)** Result of sequencing of MEN1 gene of the proband. The red arrow indicates the mutation of c.1378C>T (p.Arg460Ter) in exon 10. **(D)** The genetic locus of the son of proband. **(E)** Non-contrast-enhanced MRI scan of pituitary with a slight decrease of the T1 signal of posterior pituitary. **(F)** CT scan of adrenal gland showing bilateral nodular enlargement.

A 99mTc-methoxyisobutylisonitrile (MIBI) scan of the parathyroid showed three focal uptakes (one behind the left lobe of thyroid with the size of 34×25mm and two behind the right lobe of thyroid with the size of 10×10mm and 14×13mm, respectively), suggesting multiple parathyroid adenomas (**Figure 1A**).

Besides rehydration and potassium supplementation, the patient was treated with diuretics (furosemide) in the first 8 hours after admission. However, the serum calcium remained above 4 mmol/L. Subsequently, salmon calcitonin and bisphosphonate (zoledronic acid, 4mg, intravenous drip slowly) were given according to the guidance for Emergency management of acute hypercalcaemia in adult patients (6). The level of serum calcium gradually decreased from 4.33mmol/L to 2.37mmol/L (**Table 1**) and the eGFR maintained stable. The symptoms including thirsty, fatigue and knee soreness were relieved as well. Thereafter, the patient received subtotal parathyroidectomy (SPTX) and 3 glands were removed. Calcium (Calcium carbonate D3 tablets, 1800 mg/day) and vitamin D (Alfacalcidol Capsules, 0.75 ug/day) were supplemented after surgery (7). Meanwhile, the potassium replacement was stopped.

Postoperative pathological findings confirmed multiple parathyroid adenomas (**Figure 1B**). PTH levels decreased to normal range 3 days after surgery (**Table 1**). We re-evaluated the functions of endocrine glands after surgery. As shown in **Table 2**, cortisol level returned to normal range immediately after parathyroidectomy. The 2-day high-dose dexamethasone suppression test (HDDST, 2-mg dexamethasone every 6 hours) was performed and a suppression rate of serum cortisol more than 50% was observed (**Table 2**) (8). The concentrations of renin and aldosterone in addition to aldosterone-renin ratio (ARR) were tested after parathyroidectomy when the corresponded potassium level was normal, and no abnormal results were found. However, hemoglobin dropped to 73 g/L. After ruling out the cause of massive intraoperative blood loss and blood diseases, we suspected this drop may be attributed to the usage of zoledronic acid (9).

Because of the multiple parathyroid glands involvement, a DNA sequencing of MEN! Gene was performed. A heterozygous C to T change was identified at codon 460 in exon 10 according to the current human reference genome (GRCh37) (**Figure 1C**), which suggested a pathogenic mutation. Thus, this patient was diagnosed with MEN1. Subsequently, the MEN1 gene of her son was also evaluated, and it was identified as wild type at this genetic locus (**Figure 1D**).

Radiological screening tests for MEN1-associated tumors were conducted at the meanwhile. Considering the eGFR of the patient, the non-contrast-enhanced magnetic resonance imaging (MRI) of pituitary was performed, which showed a slight decrease of the T1 signal of posterior pituitary (**Figure 1E**). The non-contrast-enhanced adrenal computed tomography (CT) scan demonstrated bilateral nodular enlargement, considering as hyperplasia or adenoma (**Figure 1F**). There were no abnormalities observed from CT of lung, pancreas, or gastrointestinal tracts, except for nephrolithiasis in both kidneys (**Supplementary Figure 2A**) and punctate high-density shadows in the left breast (**Supplementary Figure 2B**). Breast ultrasound and mammography were not conducted because of the objection of the patient.

The patient came for the first follow-up visit two and half months after surgery. Calcium and vitamin D were regularly taken with the unchanged dosage while potassium replacement has been stopped since the operation. As shown in **Tables 1**, **2**, PTH level increased again (219.60 pg/mL) but serum calcium levels along with ACTH, cortisol, potassium, renin and aldosterone concentrations were within normal range. It was noteworthy to mention that the PTH on the second follow-up visit 4.5 months after surgery decreased to 153.50 pg/mL with serum calcium 2.31 mmol/L (**Table 1**).

## Discussion

MEN1 is a rare autosomal dominant hereditary tumor syndrome caused by a germline mutation on chromosome 11q13 (3). MEN1 gene is a tumor suppressor gene, encoding the protein menin, which plays a role in regulating gene expression and cell proliferation through selectively mediate chromatin remodeling (2).

Patients with MEN1 can present with a wide variety of manifestations including PHPT, pituitary tumor, adrenal lesion, lipoma, myoma of uterus, gastroenteropancreatic neuroendocrine tumors (GEP-NET), and breast cancer (**Table 3**), among which PHPT is one of the most frequent presentations (10). In this case, the patient presented variable clinical manifestations including bilateral enlargements of adrenal glands, mammary mass, uterine leiomyoma, and transient hypercortisolism, in addition to multiple parathyroid adenomas.

**Table 3:** Clinical concomitant manifestations of MEN 1.

MEN1-related lesion	Mean age 1 (years)	Mean age 2 (years)	n/included MEN1(%)	First manifestation (%)	Malignancy n (%)	Functional endocrine gland n (%)	Reference
PHPT	38.6 ± 14.9	45.1 ± 18	405/436 (93%)	291 (67%)	/	405 (100%)	(10)
	/	/	32/33 (96.9%)	/	/	32 (100%)	(11)
	/	/	19/20 (95%)	/	/	/	(12)
	/	/	41/49 (83.7)	/	/	/	(13)
	39	/	8/9 (89%)	4 (44.4%)	/	/	(14)
Pituitary tumor	33.4 ± 14.7	38.7 ± 15.7	178/436 (41%)	56 (12.8%)	/	142 (80%)	(10)
	/	/	16/33 (48.5%)	/	/	/	(11)
	/	/	9/20 (45%)	/	/	7 (77.8%)	(12)
	/	/	20/49 (40.8%)	/	/	/	(13)
Adrenal lesion	40 ± 4	42 ± 4	15/436 (3.4%)	2 (0.5%)	/	2 (14%)	(10)
	/	/	12/33 (37%)	/	/	/	(11)
	/	/	7/20 (35%)	/	/	0 (0%)	(12)
	45	39.6	18/67 (26%)	/	4(22.2%)	8 (44.4%)	(15)
	42.7	35.8	21/38 (55%)	/	1(4.7%)	3 (14.3%)	(16)
	/	/	30/49 (61%)	/	/	2 (6.7%)	(13)
	52.2	35.9	9/16 (56.3%)	/	/	2 (12.5%)	(14)
	46.1 ±1.4	/	146/715 (20.4%)	9 (1.2%)	10(13.8%)	11 (15.3%)	(17)
Lipoma	45	52	130/436 (30%)	1 (0.25%)	/	/	(10)
	/	/	4/20 (20%)	/	/	/	(12)
Myoma of uterus	34	48	2/5 (40%)	/	0	/	(18)
GEP-NET	37.3 ± 14.5	44.6 ± 16.1	230/436 (53%)	81 (18.6%)	/	94 (41%)	(10)
	/	/	24/33 (72.7%)	/	/	/	(11)
	/	/	20/20 (100%)	/	/	/	(12)
Breast cancer	48 ± 8.8	/	44/865 (5.1%)	/	44 (100%)	/	(4)
	/	/	1/20 (5%)	/	1 (100%)	/	(12)

Mean age 1, the mean age at diagnosis of MEN1-related lesion; Mean age2, the mean age at the diagnosis of MEN1; MEN-1, multiple endocrine neoplasia type 1; PHPT, primary hyperparathyroidism; GEP-NETs, gastroenteropancreatic neuroendocrine tumors; /, not reported.

PHPT most commonly manifests a single benign parathyroid adenoma (80%). Multiglandular disease is only seen in approximately 15%-20% of patients (19). One observational study reported that multiple adenomas or hyperplasia developed in only 7% of PHPT patients (20). On the contrary, the probability of two or more abnormal parathyroids are significantly higher in MEN1-associated PHPT (56%) (20). Thus, multiple parathyroid adenomas in this PHPT patient raised our concerns on the diagnosis of MEN1.

The adrenal lesions including cortical adenomas, hyperplasia, multiple adenomas, nodular hyperplasia, cysts, or carcinomas, are also commonly seen in MEN1 patients (**Table 3**), the percentage of which ranged from 3.4% to 61% (10–17). However, hormonal hypersecretion is rare and most of the lesions are nonfunctional (17). Importantly, nonfunctional adrenal tumors in MEN1 patients may develop into hypersecretion carcinoma (16). Waldmann J et al. reported that one in twenty-one MEN1 patients with nonfunctional adrenal tumor developed cortisol and testosterone-secreting adrenocortical carcinomas within 9 months (16). The analysis of 24 published studies covering more than 2500 cases of adrenal incidentaloma, showed a 0.1% pooled risk of developing malignancy (21). In the current case, although bilateral adrenal glands were both enlarged, levels of adrenal hormones including renin, aldosterone, metanephrine and normetanephrine were normal. Interestingly, the cortisol level was elevated along with impaired circadian rhythm before parathyroidectomy and it could not be inhibited by 1-mg overnight DST (cutoff value: serum cortisol >1.8 μg/dL) (5). Howbeit, the increased serum cortisol rapidly returned to normal range after the operation (**Table 2**), indicating a transient hypercortisolism. Possibly, the activation of adrenal cortical function in this case may be caused by the chronic condition of PHPT. However, a close follow-up of adrenal glands is recommended.

Similar to this study, the transient hypercortisolism along with increased ACTH was also reported on in a patient with PHPT other than MEN1 (22). In an observational study conducted by Rajput et al., patients with PHPT also presented loss of circadian rhythm while their plasma ACTH and morning serum cortisol were in normal range (23). The structural similarity between 15-25 amino acid of PTH and 1-11 amino acid of ACTH (24) enables PTH in high concentration to stimulate the cortisol secretion (25). This assumption is further supported by an *in vitro* experiment, in which PTH and PTH-related peptide stimulated the secretion of cortisol from dispersed human adrenocortical cells, through adenylate cyclase (AC)/protein kinase A (PKA)- and phospholipase C (PLC)/protein kinase C (PKC)-dependent signaling pathways (26). Besides PTH, calcium is also able to exert an influence on ACTH and cortisol release (27). A human study by Fuleihan et al. suggested that an calcium infusion may result in an increase of baseline ACTH levels (28). Based on these studies, the transient ACTH-independent hypercortisolism in the current case may be attributed to the increased levels of PTH and calcium (23). Nevertheless, the fluctuation of cortisol level and even the false-positive results of the 1 mg DST may also happen because of aging, hospitalization, psychiatric and stress (29). Further investigations in the pathological mechanisms and related cohort studies are necessary to disclose the root cause of the transient fluctuation in cortisol level in patients with MEN1.

There are several case reports identifying the hypokalemia in patients with PHPT (30–32) though the underlying pathogenesis was not clear. One mechanism assumption is based on renin-angiotensin-aldosterone system (RAAS) although the relationship between PTH and RAAS is still under debate. It has been reported that PTH and calcium can trigger the secretion of aldosterone *in vitro* as well as in animal models (26, 33, 34). On the contrary, a study included patients with PHPT before and after surgery demonstrated that PTH was weakly correlated with plasma renin activity but had no correlation with serum aldosterone (35). More recently, Maniero et al. showed a highly significant increase in the number of cases of HPT among patients with confirmed primary hyperparathyroidism (PA) (36), thus suggesting a bi-directional link between the adrenocortical zona glomerulosa and the parathyroid gland. The limitation in the current case is the lack of the preoperative values of renin and aldosterone. Nevertheless, it can be speculated that the hypokalemia of this patient may be related to the increased PTH and calcium levels, since the blood potassium could gradually return to normal without any potassium supplementation after parathyroidectomy.

Both *in vitro* and *in vivo* preclinical studies suggest that *MEN1* gene is implicated to the occurrence and development of breast cancer (37, 38). Several case reports (39) and human observational studies also support the conclusion that female MEN1 patients suffer increased risk for breast cancer, the standardized incidence ratio of which is ranged from 1.96 to 2.14 (4). A mammary mass was identified in this case (**Supplementary Figure 2**). Although the patient refused further examinations, cancer surveillance was recommended due to the potential risk of breast cancer in subjects with MEN1. In addition, McKeeby et al. reported the potential relationship between uterine leiomyoma and MEN1. Five of six uterine leiomyomata in two patients with MEN1 exhibited 11q13 loss of heterozygosity (LOH), indicating that smooth muscle tumors of uterus in MEN1 patients may develop through the inactivation of MEN1 gene (18).

According to the clinical practice guideline for MEN1 (3), a diagnosis of MEN1 may be established based on one of three criteria, defined from clinical, familial and genetic perspectives. MEN1 mutational analysis should be taken under the following situations: 1) an index case with two or more MEN1-related endocrine tumors; 2) first-degree relatives of an MEN1 mutation carrier; 3) in patients with suspicions or atypical for MEN1 (3). The last situation with multiple parathyroid glands involvement is an indication for MEN1 mutation testing (3), which might be neglected due to insufficient knowledge of MEN1. In the current case, although there were no evidence suggesting that the patient’s first-degree relatives were MEN1 mutation carriers, MEN1was still highly suspicious because of multiple parathyroid adenomas. Accordingly, a genetic testing was performed and a mutation of c.1378C>T (p.Arg460Ter) in exon 10 was identified, which has been previously reported in MEN1 patients (40).

The therapeutic strategy was similar to that of the specific tumors in non-MEN1 patients. The MEN1 guideline (3) recommend 3.5 glands SPTX or total parathyroidectomy (TPTX) for MEN1-related PHPT; however, no clear conclusion on which option is better, considering the recurrent rate and hypoparathyroidism (41–43). In addition, one should note that the treatment effect in MEN1 patients may not be comparable to that of non-MEN1 patients, because multiple endocrine tumors may be larger, more aggressive, and poorly respond to the treatment. It has been reported that MEN1-related PHPT has a higher recurrence rate compared to PHPT in non-MEN1 patients (40-60% versus 4-16%) (3). Thus, periodic clinical surveillance is required, including biochemical test and imaging screening.

### Limitation

The patient had hysterectomy a long time ago and we cannot verify the MEN1 mutation in her fibroid. Additionally, preoperative renin and aldosterone were not tested immediately because of the patient’s poor health conditions and hypokalemia. Lack of these values makes it difficult to determine the role of RAAS in the pathogenesis of hypokalemia in the current case. Meanwhile, a lesion in left breast was identified during her hospitalization, but this patient refused further examination to evaluate the possibility of breast cancer.

## Conclusion

In this case of PHPT, multiple parathyroid adenomas draw our attentions, which prompted us to test MEN1 gene mutation and to screen for other neuroendocrine tumors (NETs). The transient hypercortisolism may present in MEN1-associated PHPT, and return to normal after parathyroidectomy, along with decreases in serum calcium and PTH. In addition, MEN1 related tumors may grow at any time and convert from non-functional tumor to malignancy. Accordingly, follow-up with the biochemical and imaging screening for endocrine organs should be performed periodically and closely.

## Data Availability Statement

The original contributions presented in the study are included in the article/**Supplementary Material**. Further inquiries can be directed to the corresponding author.

## Ethics Statement

This study was approved by the Ethics Committee of Tongji Hospital, Huazhong University of Science & Technology. Written informed consent was obtained from the individuals for the publication of any potentially identifiable images or data included in this article. Written informed consent was obtained from the individual(s) for the publication of any potentially identifiable images or data included in this article.

## Author Contributions

All the authors have contributed significantly. FC collected the clinical data, wrote the manuscript. QX summarized the relevant literature. WY and XY give suggestions about clinical investigations. All the work was done under the instructions of SS. All authors contributed to the article and approved the submitted version.

## Conflict of Interest

The authors declare that the research was conducted in the absence of any commercial or financial relationships that could be construed as a potential conflict of interest.

## Publisher’s Note

All claims expressed in this article are solely those of the authors and do not necessarily represent those of their affiliated organizations, or those of the publisher, the editors and the reviewers. Any product that may be evaluated in this article, or claim that may be made by its manufacturer, is not guaranteed or endorsed by the publisher.

## Supplementary Material

Supplementary Figure 1 The timeline of hospitalization and follow-up visits.

Supplementary Figure 2
**(A)** Renal CT scan. **(B)** Chest CT scan.
