# Supplementary figures and images for: A MEN1 Patient Presenting With Multiple Parathyroid Adenomas and Transient Hypercortisolism: A Case Report and Literature Review
Source: Front Endocrinol (Lausanne). 2022 Mar 8;13:802453. doi: 10.3389/fendo.2022.802453 (PMC8965320; doi:10.3389/fendo.2022.802453)

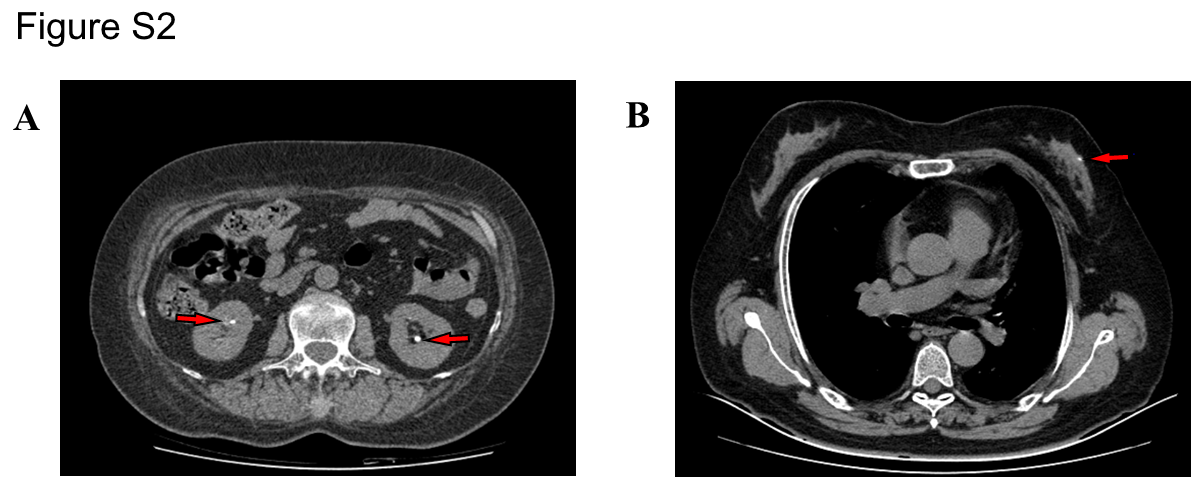

Supplement: Supplementary Figure 2 — (A) Renal CT scan. (B) Chest CT scan. [file Image_2.tiff]
